# Changes in aspects of social functioning depend upon prior changes in neurodisability in people with acquired brain injury undergoing post-acute neurorehabilitation

**DOI:** 10.3389/fpsyg.2015.01368

**Published:** 2015-09-08

**Authors:** Dónal G. Fortune, R. Stephen Walsh, Brian Waldron, Caroline McGrath, Maurice Harte, Sarah Casey, Brian McClean

**Affiliations:** ^1^Centre for Social Issues, Department of Psychology, University of LimerickLimerick, Ireland; ^2^Acquired Brain Injury IrelandDun Laoghaire, Ireland

**Keywords:** acquired brain injury, rehabilitation, mental health, disability, participation, QoL, prospective study

## Abstract

Post-acute community-based rehabilitation is effective in reducing disability. However, while social participation and quality of life are valued as distal outcomes of neurorehabilitation, it is often not possible to observe improvements on these outcomes within the limited time-frames used in most investigations of rehabilitation. The aim of the current study was to examine differences in the sequence of attainments for people with acquired brain injury (ABI) undergoing longer term post-acute neurorehabilitation. Participants with ABI who were referred to comprehensive home and community-based neurorehabilitation were assessed at induction to service, at 6 months and again at 1.5 years while still in service on the Mayo-Portland Adaptability Index (MPAI-4), Community Integration Questionnaire, Hospital Anxiety and Depression Scale, and World Health Organisation Quality of Life measure. At 6 months post-induction to service, significant differences were evident in MPAI abilities, adjustment, and total neurodisability; and in anxiety and depression. By contrast, there was no significant effect at 6 months on more socially oriented features of experience namely quality of life (QoL), Community Integration and Participation. Eighteen month follow-up showed continuation of the significant positive effects with the addition of QoL-related to physical health, Psychological health, Social aspects of QoL and Participation at this later time point. Regression analyses demonstrated that change in QoL and Participation were dependent upon prior changes in aspects of neurodisability. Age, severity or type of brain injury did not significantly affect outcome. Results suggest that different constructs may respond to neurorehabilitation at different time points in a dose effect manner, and that change in social aspects of experience may be dependent upon the specific nature of prior neurorehabilitation attainments.

## Introduction

Acquired brain injury (ABI) is the leading cause of death and disability in young people aged 18–24 years: it also disproportionately affects children (up to 4 years-old) and people aged over 65 years (Yates et al., 2006). ABIs can result from a number of causes including traumatic brain injury [including road traffic accidents, assault or falls, cerebrovascular accidents (e.g., strokes or bleeds), or other internal processes (encephalitis, infection, anoxia, etc.)]. ABI brings with it the significant potential for life-long functional changes encompassing a range of physical, cognitive, emotional, behavioral and social changes, which mandate a process of often lengthy rehabilitation to enable the person with ABI to optimize their recovery (Turner-Stokes, 2008; Cicerone et al., 2011).

The value of specialized rehabilitation for brain injury, including community-based rehabilitation, is becoming increasingly apparent in terms of both functional outcomes (Schnitzler et al., 2014), and cost effectiveness (van Heugten et al., 2012; Oddy and Ramos, 2013). Evidence based reviews have generally reported positive outcomes of engagement in comprehensive holistic home and community-based rehabilitation programs (Turner-Stokes, 2008; Geurtsen et al., 2010; Cicerone et al., 2011; van Heugten et al., 2012), although with some exceptions depending on the outcomes assessed (Institute of Medicine, 2011; Brasure et al., 2012).

A number of features of participant’s experience have been suggested to be important targets for, and outcomes of, intervention. Changes in neurodisability is one of the most prominent outcomes and in addition to targeting changes in physical and cognitive abilities and mental health, rehabilitation programs increasingly utilize more socially moderated factors such as quality of life and participation in society (Eicher et al., 2012; Stiers et al., 2012; Altman et al., 2013; Malec et al., 2015). Such social/community integration factors are becoming important features of outcome (Haslam et al., 2008; Cicerone et al., 2011; Algurén et al., 2012; Brasure et al., 2012; Stalder-Lüthy et al., 2013; Walsh et al., 2015).

Mental health difficulties, particularly anxiety and depression have relatively common currency in ABI and pose a significant barrier to rehabilitation progress (Gould et al., 2011; Bertisch et al., 2013; Stalder-Lüthy et al., 2013). Indeed the odds of developing depression following brain injury are more than five times higher than in the general population (Osborn et al., 2014). A number of studies have suggested that 6 months following ABI, one third of individuals develop clinically relevant symptoms of depression (Stalder-Lüthy et al., 2013), and that in the first year, over 60% have a diagnosable psychiatric disorder, principally anxiety, and depression (Gould et al., 2011). While it has been suggested that rehabilitation may improve psychological difficulties (Geurtsen et al., 2010; Stalder-Lüthy et al., 2013), inadequate follow-up has hampered the information that can be derived from such studies (Brown et al., 2011). It is compelling that a recent meta-analysis of psychological interventions for depression following ABI expressed “amazement” at the small number of published studies available for analysis despite the high prevalence of mental health difficulties in ABI (Stalder-Lüthy et al., 2013).

Community participation and quality of life (QoL) are increasingly valued as neurorehabilitation outcomes (Cicerone et al., 2011). Participation has much in common with social and community reintegration and relates to acceptable levels of function in social roles or relationships (Whiteneck et al., 2011). The most recent review specifically examining participation outcomes reported that the available evidence was too limited to draw robust conclusions about the effects of neurorehabilitation on participation (Brasure et al., 2013). Thus while participation and indeed QoL are important pragmatic outcomes in rehabilitation, relatively brief periods of intervention, limited opportunity to address the application of interventions to everyday functioning, lack of follow-up assessing community functioning, and failure to include relevant outcome measures has led to limited data in this area (Cicerone et al., 2011).

In terms of QoL, Geurtsen et al. (2011), reported improvements in QoL, societal participation and community integration, and emotional well-being that were maintained in 96% of cases at 1 year follow-up, but did not increase at 3 years follow-up (Geurtsen et al., 2012). In people with cerebrovascular accident (CVA), Algurén et al. (2012) reported that in the first 3 months of rehabilitation, body functions, activities, and participation explained the majority of the variance in participants’ QoL. At 1 year, only body functions and environmental factors accounted for significant variance in QoL. This difference in outcome across time raises the question as to whether a dose effect of rehabilitation duration significantly affects outcome or whether a sequence effect is evident with certain prior attainments needing to be set in place to bolster subsequent changes in these outcomes.

There is some recent evidence supporting a dose-effect relationship on outcome of neurorehabilitation. In a large study of a cohort of people with CVA (Altman et al., 2013), participants who completed their full neurorehabilitation program – what the authors term a full dose – had improved outcomes in terms of neurodisability when compared with those who were precipitously discharged and thus did not complete their full program. A dose effect of multidisciplinary intervention would seem plausible and indeed in terms of cortical plasticity, Kolb and Muhammad (2014), make the point that an effective treatment for individuals after brain injury would have to be intense, regular, and interdisciplinary including cognitive, behavioral, social, and physical/biological aspects of intervention. While there are a number of studies examining intake factors that may predict rehabilitative outcome (van Heugten et al., 2012; Hayden et al., 2013; Snell et al., 2013), there is a paucity of studies that have addressed the effects of participants’ prior attainment of within-program outcomes on subsequent attainment of further outcomes. Moreover, a recent landmark review of evidence based cognitive rehabilitation (Cicerone et al., 2011), makes the point that although social participation and quality of life are valued as the distal health-related outcomes of neurorehabilitation, it is often not possible to observe improvements on these outcomes within the limited time-frames used in most investigations of neurorehabilitation.

The objectives of the current study were therefore, firstly to examine whether participants demonstrate significant improvement on a broad number of domains assessed at shorter and longer durations of community-based neurorehabilitation: namely neurodisability, community integration, mental health, and quality of life. Secondly to examine the contribution of differences in clinical features of injury such as age at onset, injury severity, type and duration of brain injury, age, and sex on changes from pre-treatment to follow-up. Thirdly to investigate whether changes in the more socially oriented factors including QoL, participation and social/community integration occurred later than changes in neurodisability and mental health outcomes and furthermore whether such changes in QoL, participation and community integration may be dependent upon prior changes in neurodisability and mental health outcomes.

## Materials and Methods

### Participants

Eighty three people were eligible for participation in the cohort study. A total of six participants dropped out of the study between their initial induction assessment and follow-up assessment. Given that there was only one significant difference between the induction data for these six participants and the cohort (with people who dropped out reporting marginally lower quality of life related to physical health at induction – Mann–Whitney *U* = 118.5, *p* = 0.047), for parsimony, these six participants were excluded from further analysis. The final cohort consisted of 77 participants who were assessed at induction to service (time 1), 6 months later (time 2) and at 1.5 years post-induction (time 3). In addition to the cohort of participants followed up over three time points during their service, a total of 151 additional people with ABI who were referred to service after the cohort had been established, during this 2 years period were assessed at induction to the service for comparison purposes with all induction data of the cohort. The purpose of this cross sectional sample was to examine goodness of fit of the cohort to those routinely referred to post-acute neurorehabilitation services.

Inclusion criteria for participation in this study were age > 18 years, clinical confirmation of ABI specifying acquired non-progressive injury to the brain, onset of ABI < 65 years, and sufficient proficiency in English to undertake the study. All participants were engaged in an individualized Home and Community Based Rehabilitation program accredited by the Commission for Accreditation of Rehabilitation Facilities (CARFs).

### Procedure

The protocol was approved by the ABI Ireland national research ethics committee and all participants provided full consent for participation. Assessments were administered by staff as semi structured interviews. All clients of the service were eligible for participation where the inclusion criteria was deemed appropriate by members of the Clinical Service Team.

### Measures

Demographic, clinical and social information was recorded at induction to the study (**Table 1**).

**Table 1:** Demographics, injury characteristics, and clinical features of cohort.

**Gender**	
Male *n* %	51 (66%)
Female	26 (34%)
Age years mean *SD*	47.19 (12.8)
Duration ABI (years)	10.02 (8.4)
Age at onset (years)	37.80 (15.3)
**Cause of injury n %**	
Traumatic brain injury	37 (48%)
Cerebrovascular accident	33 (43%)
Tumor	3 (4%)
Anoxia	3 (4%)
Encephalitis	1 (1%)
**Severity of ABI**	
Moderate	33 (43%)
Severe	44 (57%)

#### Injury Severity

Injury severity in TBI was calculated using the standard assessments of severity using Glasgow Coma Scale (GCS) scores, duration of Post-Traumatic Amnesia, and Loss of Consciousness (LOC) using the following procedure: Severe Brain Injury = GCS score less than 9, LOC longer than 24 h, or PTA longer than 1 week. Moderate Brain Injury = GCS score of 9–12, LOC of 30 min to 24 h, or PTA of 24 h to 1 week. Mild Brain Injury = GCS score higher than 12, LOC less than 30 min, or PTA less than 24 h. If more than one indicator was present and differed in level of severity, the more severe level was assigned. For other causes (e.g., CVA, encephalitis, anoxia, and tumor), severity was assessed by multidisciplinary team discharge report from acute (hospital based) rehabilitation services specifying moderate and severe disability.

Participants completed the following measures of Neurodisability, Community Integration, Mental Health, and Quality of Life at induction to post-acute neurorehabilitation, at 6 months follow-up and at 1.5 years post-induction.

#### NeuroDisability – Mayo Portland Adaptability Inventory – (MPAI-4)

The MPAI-4 is a widely used measure of limitations imposed by brain injury (Malec, 2004; Malec and Lezak, 2008). The measure yields a total score reflecting overall disability, as well as three subscale scores for the Ability Index (e.g., mobility, cognitive functioning, communication), Adjustment Index (e.g., pain, mood, fatigue), and Participation Index (e.g., social contact, independent living, employment). Prior studies have demonstrated satisfactory internal consistency and construct validity, as well as concurrent and predictive validity, for the full measure and its indices (Wilde et al., 2010; Kean et al., 2011; Malec et al., 2012). The MPAI-4 has been shown to be sensitive to clinical change in studies of rehabilitation interventions (Eicher et al., 2012), and that all 30 items could be mapped to components and categories in the WHO-ICF (Lexell et al., 2012). In the current study, internal consistency was good for the MPAI total scale score (0.91), Abilities (0.74), Adjustment (0.82), and Participation Indices (0.85).

#### Community Integration Questionnaire

The Community Integration Questionnaire (CIQ; Corrigan and Deming, 1995; Salter et al., 2011), is a brief assessment of community integration that comprises 15 items assessing effective role performance in three domains: home integration (active participation in the operation of the home or household), social integration (participation in social activities outside the home) and productivity (regular performance of work, school or volunteer activities). Internal consistency in previous studies has been reported as good, with Cronbach’s alpha’s ranging from 0.76 to 0.84 for total scale scores (Corrigan and Deming, 1995). The CIQ is predominately linked to the major life areas (35%), community, social and civic life (31%), and domestic life (19%) chapters of the WHO ICF (Salter et al., 2011). A measure of Minimal Clinically Important Difference (MCID) of 4.2 CIQ points has been provided for the CIQ (Cicerone et al., 2004). Internal consistency was good in the current study for CIQ total score (0.71) and Home integration subscale (0.83), but was unacceptably low for Social integration (0.45), and Productivity (0.23). As such it was decided not to use the Social Integration and Productivity subscales further in the analyses.

#### Hospital Anxiety and Depression Scale

The Hospital Anxiety and Depression Scale (HADS; Zigmond and Snaith, 1983), a 14-item measure, was used to assess symptoms of anxiety and depression. Items are rated on a 0–3 point scale indicating the strength of agreement with each item. Thus, scores for each subscale range from 0 to 21. It has been widely used in studies with patients with brain injury and has been shown to be an appropriate measure of anxiety and depression and of distress more generally (Dawkins et al., 2006; Schönberger and Ponsford, 2010). A score of >7 on either scale indicates the presence of clinically relevant distress. In the current study, the anxiety and depression subscales yielded good internal consistency scores (0.79 and 0.76 respectively).

#### World Health Organisation’s Quality of Life Scale (WHOQoL- BREF)

The WHOQoL-BREF is a 26 item international cross-culturally comparable quality of life assessment instrument. The assessment examines a person’s Quality of Life in relation to four domains: QoL related to Physical Health, Psychological Health, Social Relationships, and the person’s living Environment. Higher scores denote better QoL. The measure has demonstrated appropriate reliability and validity (WHOQOL Group, 1998; Skevington et al., 2004), and has been used successfully in people with ABI (Chiu et al., 2006; Polinder et al., 2015). In the current study, internal consistency was good for the QoL subscales of Physical Health (0.72), Psychological Health (0.78), and environmental aspects of QoL (0.79). While the Social aspects of QoL subscale was somewhat lower (0.61), it was decided to retain this particular subscale as the alpha was more likely due to the small number of items in the subscale rather than problem in psychometrics (e.g., intercorrelations between items were good).

### Statistical Analysis

Descriptive statistics were computed for variables relating to injury and demographics. Q-Q plots and Kolmogorov–Smirnov test were used to examine the distribution of outcome data. Given that outcome data showed no significant deviation from normality (*Z*’s > 1.21, *p*’s > 0.14), means and standard deviations were calculated for the four main outcome measures and subscales as appropriate. Repeated measures analysis of variance models were used to model the means of each of the outcome measures over time. Effect sizes [Partial eta squared ($\eta_{p}^{2}$)] were considered small when between 0.5 and 0.10, medium when between 0.10 and 0.20, and large when greater than 0.20. *Post hoc* pairwise comparisons were conducted and a Bonferroni adjustment (α = 0.004) was performed to examine differences between outcomes at time 1 and time 2, and between time 2 and time 3. Categorical data were analyzed by Chi square test and longitudinal categorical data by Cochrane’s *Q*-test for three time points and the McNemar test for two time points. Repeated measures ANCOVA was used to examine differences in outcome for the two principal causes of ABI; injury resulting from an external force (TBI), or injury resulting from an internal disease process (CVA, encephalitis, hypoxia, or tumor), and severity of injury. Zero order correlations and multiple regression analysis were used to examine the potential influence of prior changes in neurodisability and mental health on subsequent changes in QoL and Participation.

## Results

**Table 1** details demographics, injury characteristics and clinical features of the sample. Participants were predominantly male, and TBI was the most common mechanism of injury, chiefly resulting from road traffic accidents and falls. The majority of participants had a severe brain injury.

### Comparison of the Induction Data of the Cohort with Induction Data of Referrals within the Lifetime of the Study

There were no significant differences between the cohort (*N* = 77) and people who were referred for service in the 2 years of the study (*N* = 158) on any of the outcome assessments at induction (*t*_226_ < 2.31, *p* > 0.02). There were also no significant differences between the cohort and single assessment groups on participants’ age, age at onset of their ABI, or duration with ABI (*t*’s_215_ < 2.27, *p* > 0.03), or on clinical severity of their injury (χ^2^ = 2.35, *p* = 0.13). There was also no significant difference between the cohort and people who completed their assessments at induction on numbers of people with CVA vs. TBI (χ^2^ = 0.71, *p* = 0.41). The cohort was therefore not unrepresentative of the profile of people with ABI routinely referred to post-acute neurorehabilitation services in the Republic of Ireland.

### Demographic Effects on the Cohort at Induction

At induction to the study, cohort participants’ performance on neurodisability, community integration, distress and QoL did not differ as a function of gender (*t*’s < 2.24, *p* > 0.03), participants’ age, duration with ABI, or age at onset of their injury (*r*’s < 0.27, *p* > 0.03).

Independent *t*-tests (α < 0.004) demonstrated that when compared to participants with mild/moderate injury, people who had sustained a severe brain injury were functioning at a poorer level at induction in terms of the MPAI assessment of Abilities (*t*_77_ = -4.40, *p* = 0.001), Adjustment (*t*_77_ = -4.08, *p* = 0.001), and Participation (*t*_77_ = -5.27, *p* = 0.001). Whether a person sustained a moderate or severe brain injury did not significantly affect scores on Community Integration, Mental Health or QoL at induction (*t*’s_77_ < 1.50, *p* > 0.0.13). Means and standard deviations for all outcome measures are presented in **Table 2**.

**Table 2:** Mean (*SD*) of measures at induction and follow-up time points.

Measure	Induction mean	6 months follow-up mean	1.5 years follow-up mean
**Neurodisability**	
MPAI-4 total scale score	43.83 (21.97)	40.09 (20.93)	33.70 (17.91)
MPAI abilities	15.15 (8.30)	13.49 (7.85)	12.11 (7.03)
MPAI adjustment	15.61 (9.71)	13.35 (8.38)	10.63 (7.10)
MPAI participation	13.06 (7.40)	13.41 (7.49)	11.05 (6.73)
**Community integration**	
CIQ total score	14.48 (5.04)	15.29 (5.13)	15.39 (5.35)
CIQ home integration	4.24 (3.08)	4.65 (3.15)	4.76 (3.29)
**Mental health – HADS**	
HADS anxiety	5.95 (4.08)	4.96 (3.21)	4.28 (3.46)
HADS depression	6.16 (4.22)	5.12 (3.27)	4.56 (3.33)
**Quality of life – WHOQoL-Bref**	
Physical QoL	12.66 (2.04)	12.73 (2.05)	13.38 (1.91)
Psychological QoL	12.61 (2.16)	12.90 (2.20)	13.50 (2.29)
Social QoL	13.17 (3.77)	13.31 (3.72)	14.03 (3.76)
Environmental QoL	15.25 (2.54)	15.59 (2.68)	15.79 (2.98)

### Neurodisability

A single repeated measures analysis of variance was used to model the MPAI Abilities, Adjustment, Participation, and Total scale score over the three time points. Significant increases were apparent for people with ABI in terms of their Abilities (*F*_1,76_ = 15.29, *p* = 0.001, $\eta_{p}^{2}$ = 0.17), Adjustment (*F*_1,76_ = 36.87, *p* = 0.001, $\eta_{p}^{2}$ = 0.33), Participation (*F*_1,76_ = 19.33, *p* = 0.001, $\eta_{p}^{2}$ = 0.20), and total Neurodisability (*F*_1,76_ = 33.82, *p* = 0.001, $\eta_{p}^{2}$ = 0.31). To permit comparisons with previously published studies, the standardized T score for the total MPAI was 48.58 at induction and 39.81 at the final assessment time-point (lower scores = better outcomes).

Pairwise comparisons revealed significant induction to 6 months follow-up improvements for Abilities (*t*_76_ = 3.11, *p* = 0.003), Adjustment (*t*_76_ = 4.44, *p* = 0.001), and total Neurodisability (*t*_76_ = 3.12, *p* = 0.003), but not for Participation (*t*_76_ = 0.93, *p* = 0.35).

Significant pairwise comparisons of 6 months to 1.5 years data were found for Adjustment (*t*_76_ = 4.82, *p* = 0.001), Participation (*t*_76_ = 5.97, *p* = 0.001), and total Neurodisability (*t*_76_ = 5.27, *p* = 0.001). However, changes in Abilities failed to reach significance (*t*_76_ = 2.44, *p* = 0.01).

Given that participants with severe brain injury performed more poorly than participants with moderate brain injury in terms of their MPAI performance at induction, an adjusted model was fit to the data which included an interaction effect for time by severity of ABI. The interaction term was significant for Abilities (*F*_1,75_ = 7.09, *p* = 0.002, $\eta_{p}^{2}$ = 0.08) and total scale score (*F*_1,75_ = 5.90, *p* = 0.003, $\eta_{p}^{2}$ = 0.07), but not for Participation (*F*_1,75_ = 1.91, *p* = 0.15, $\eta_{p}^{2}$ = 0.02), or Adjustment (*F*_1,75_ = 2.64, *p* = 0.07, $\eta_{p}^{2}$ = 0.03). Effect sizes were small.

### Community Integration

A single repeated measures analysis of variance was used to model the total Community Integration scale score and Home Integration scores over time. Neither Home Integration (*F*_1,75_ = 2.21, *p* = 0.09, $\eta_{p}^{2}$ = 0.03), or total Community Integration (*F*_1,75_ = 2.58, *p* = 0.07, $\eta_{p}^{2}$ = 0.03) showed statistically significant improvements over time.

Using published MCID scores (Cicerone et al., 2004), for the total scale score of the CIQ (MCID = 4.2), 16% of participants had achieved the MCID score at 6 months, with 35% achieving it at 1.5 years. McNemar’s test demonstrated that this change from time 2 to time 3 was significant (*p* = 0.009).

### Mental Health

#### Depression

Repeated measures ANOVA demonstrated that mean reductions in depression scores over time were statistically significant (*F*_1,75_ = 6.82, *p* = 0.001, $\eta_{p}^{2}$ = 0.09) albeit with a modest effect size. Pairwise comparisons also showed significant pre-treatment to 6 months improvements (*t*_76_ = 2.78, *p* = 0.001), however, the 6 months to 1.5 years data was not significant (*t*_77_ = 1.55, *p* = 0.12).

Using the established cut-offs for the presence of clinical distress, at induction to the study 39% of clients (*n* = 30/77) scored above the cut-off for clinically relevant depressive symptoms (HADS Depression subscale > 7). This figure had fallen to 24.6% (*n* = 19/77) after 6 months of rehabilitative intervention, and to 20% (*n* = 15), 1 year later (1.5 years post-baseline). This represented a significant effect (Cochrane’s *Q* = 11.31, df 2, *p* = 0.003). *Post hoc* McNemar test with Bonferroni correction suggested that the principal difference was between induction and 6 months follow-up only (*p* = 0.01).

#### Anxiety

Repeated measures ANOVA suggested that mean differences in anxiety over time were statistically significant (*F*_1,75_ = 9.90, *p* = 0.001, $\eta_{p}^{2}$ = 0.12). Pairwise comparisons also showed significant improvements from induction to 6 months (*t*_76_ = 3.96, *p* = 0.001) but not 6 months to 1.5 years (*t*_76_ = 3.96, *p* = 0.001). Mean (*SD*) scores are presented in **Table 2**.

In terms of clinically relevant anxiety (HADS Anxiety subscale > 7), at induction 31.2% of clients assessed (*n* = 24/77), scored above the cut-off for clinically relevant symptoms of anxiety. This figure had fallen to 14.3% (*n* = 11/77) after 6 months of rehabilitative intervention, and had increased slightly to 15.6% (*n* = 12/77) 1 year later. Cochrane’s *Q*-test suggested this represented a significant effect (Cochrane’s *Q* = 13.08, df 2, *p* = 0.001), with *post hoc* McNemar tests again finding that the significant reduction was between induction and 6 months only (*p* = 0.001).

### Quality of Life

A single repeated measures analysis of variance was used to model the Quality of Life data. Significant mean differences over time were evident for QoL related to Physical Health (*F*_1,75_ = 9.49, *p* = 0.001, $\eta_{p}^{2}$ = 0.11), Psychological Health (*F*_1,75_ = 10.31, *p* = 0.001, $\eta_{p}^{2}$ = 0.12), and Social aspects of QoL (*F*_1,75_ = 3.61, *p* = 0.03, $\eta_{p}^{2}$ = 0.05), but not environmental aspects (*F*_1,75_ = 2.66, *p* = 0.07, $\eta_{p}^{2}$ = 0.03).

Pairwise comparisons (α < 0.004) demonstrated no significant induction to 6 months follow-up improvements for any of the quality of life measures (*t*_75_ < 1.86, *p* = 0.06). Comparisons of 6 months to 1.5 years follow-up revealed significant improvements on QoL related to Physical Health (*t*_76_ = -3.31, *p* = 0.001), Psychological health (*t*_76_ = 2.83, *p* = 0.003), but not Social aspects of QoL (*t*_76_ = 2.09, *p* = 0.03).

### Prediction of Quality of Life and Participation Improvements by Prior Improvements in Neurodisability and Mental Health

We next examined whether the significant changes in QoL and Participation from 6 months to 1.5 years were dependent upon prior (induction to 6 months) changes in neurodisability and mental health.

Prior to building the regression model, correlation analysis was undertaken (**Table 3**) which suggested that significant 6 months to 1.5 years changes in Participation was related to prior improvements in the Neurodisability factors of Abilities (*r* = -0.49, *p* = 0.001) and Adjustment (*r* = -0.36, *p* = 0.01) and in prior changes in Depression (*r* = -0.31, *p* = 0.01). *t*-test showed no significant effect of injury severity on 6 months to 1.5 years Participation scores (*t*_76_ = 0.96, *p* = 0.34).

**Table 3:** Correlations between changes in disability and distress from induction to 6 months, changes in QoL and participation from 6 months to 1.5 years.

Induction – 6 months difference scores				

**6 month – 1.5 years difference scores**	**MPAI abilities**	**MPAI adjustment**	**HAD depression**	**HAD anxiety**
Physical health related QoL	-0.31^∗^	0.32^∗∗^	0.02	-0.10
Psychological Qol	0.13	0.17	-0.04	-0.12
Social QoL	0.09	-0.12	0.03	0.06
MPAI participation	-0.49^∗∗^	-0.36^∗∗^	-0.31^∗^	0.06

*^∗^*p* < 0.05, ^∗∗^*p* < 0.01*.

The regression model (**Table 4**) for change in Participation was significant for the three variables – prior improvements in Depression, Adjustment to disability and Abilities (*R*^2^ = 0.24, *F*_1,75_ = 14.49, *p* = 0.001). However, only prior improvement in Abilities predicted subsequent improvements in Participation in the final regression equation (β = 0.49, *t* = -3.81, *p* = 0.001).

**Table 4:** Prediction of improvements in participation and quality of life at 1.5 years post-induction by earlier improvements in neurodisability and mental health.

Measure	Predictors	*B*	*p*	95% CI
**Participation**				
	Abilities	-0.49	0.001	-0.82 to -0.02
	Depression	-0.22	0.09	-0.64 to 0.05
	Adjustment	0.06	0.77	-0.32 to -0.42
**Physical health related QoL**				
	Adjustment	-0.32	0.01	-0.23 to -0.03
	Abilities	0.04	0.85	-0.16 to -0.19

The significant improvement in QoL related to Physical Health at 1.5 years follow-up was associated with prior significant changes from baseline in Abilities (*r* = -0.31, *p* = 0.01) and Adjustment (*r* = -0.32, *p* = 0.01). Neither changes in QoL related to psychological health nor social aspects of QoL were associated with prior changes in neurodisability or mental health (*r*’s < 0.17). The regression model for positive change in QoL-related Physical health at time 3 was initially significant for prior improvements in both Abilities and Adjustment (*R*^2^ = 0.10; *F*_1,75_ = 6.26, *p* = 0.01). However, improvements in QoL-related Physical Health was solely predicted by prior improvements in MPAI Adjustment only (β = -32, *t* = -2.50, *p* = 0.01) in the final equations (**Table 4**).

## Discussion

This study has presented data on a prospective cohort of longer-term individuals with moderate to severe brain injury in continuous service at 6 months and 1.5 years on a range of measures that are a common focus of outcome assessment and goal setting with people with ABI. In view of the need to provide outcome information across a broad domain of functioning including physically oriented, social/community oriented, and well-being outcomes, this study has added important additional information on the level of improvement and differences in the rate of improvement for proximal and distal outcomes across time for people with moderate-severe brain injury.

The first two objectives of the study were to examine whether and to what extent people in receipt of home and community-based neurorehabilitation showed improvements in terms of neuro-disability, community integration, mental health and QoL over time in service, and secondly to investigate whether changes in the more distal outcomes of QoL, Community Integration and Participation occurred at a later stage than changes in more general aspects of neurodisability and mental health. To this end, participants in service showed significant improvement in terms of their cognitive and physical abilities, adjustment to brain injury, in aspects of QoL, and in anxiety and depression. While the use of continuous data did not reach significance for Community Integration, use of published MCID values (Cicerone et al., 2004) showed significant changes in the number of participants attaining Community Integration MCIDs from 6 months to 1.5 years follow-up.

Patient’s performance on measures of neurodisability, essentially participants’ Abilities and Adjustment to brain injury showed the most consistent improvements over time for the cohort. Using the MPAI-4’s standardized T scores for the total scale, the difference in T scores from induction to rehabilitation to 1.5 years follow-up was broadly similar to recent published work on comprehensive community rehabilitation for longer term cases (Altman et al., 2010; Curran et al., 2014), and indeed the difference in T scores in the current study approaches that recently published for people with duration of ABI of more than 1 year (Altman et al., 2013). This suggests a robust improvement across time on this measure in the context of people in continuing service in the current study.

The well-recognized barrier that mental health difficulties can pose to rehabilitation progress (Stalder-Lüthy et al., 2013; Merzenich et al., 2014), was also addressed as an outcome and our results suggest that the significant time for improvement of anxiety and depression is between induction and 6 months in service. Commensurate with this finding, the number of participants whose scores positioned them in the more severe anxiety and depression categories declined and consolidated over the course of the program. However it is apparent that a small but significant proportion of people (14–16%) were dealing with mental health difficulties arising from their ABI that were resistant to change and were continuing to experience on-going mental health challenges at 1.5 years follow-up. Identification and management of this particular group of participants’ demands careful assessment at induction to neurorehabilitation programs. Such assessment is required to ensure that participants are triaged toward the most effective content and duration of intervention for their particular mental health needs.

Recent studies have begun to suggest a dose-effect relationship on outcome of neurorehabilitation. Previous research in a cohort of people with CVA (Altman et al., 2013), reported that completing a planned neurorehabilitation program (a full dose) resulted in superior outcomes when compared with those who did not complete their full rehabilitation program, and suggested that that this dose effect relationship may relate to intensity, quality and appropriateness of services and not simply time in the program. The results of the current study extend these finding to the broader ABI population and also adds to this finding by providing data that suggests that the nature of the outcome is related to the dose of neurorehabilitation, but also to the nature of what prior changes have already been set in place by participation in neurorehabilitation. Our results suggest that changes in Qol and Participation were initially associated with prior changes in neurodisability and mental health. However, the final model suggested that longer-term improvements in QoL and Participation were predicted by Neurodisability factors alone. This finding suggests that change following ABI, particularly in relation to these more nuanced outcomes, may be sequenced and dependent upon the content, duration, and prior attainment of neurodisability aspects of outcome.

### Limitations

The current study is a cohort study and caution should therefore be exercised in drawing any conclusion that neurorehabilitation alone is responsible for the changes observed. The use of long-term cases, while making spontaneous improvement less likely does not remove its possibility. Further, while participants were engaged in a national Home and Community-based rehabilitation program accredited through international best practice (CARF), the participants in this study were individuals at the more severe end of the brain injury spectrum and as such results may not generalize to individuals with less severe brain injuries or with a shorter duration of injury.

## Conclusion

Nonetheless, this study has demonstrated improvements over time for participants in long-term service with moderate to severe brain injury across a range of measures of outcome. Results also suggested the presence of a dose effect which varied as a function of the nature of the outcome, with some outcomes showing the need for increased time duration, and others demonstrating consolidation after a shorter duration of intervention. Importantly, this study revealed that significant changes in more nuanced person-centered and social aspects of outcome such as quality of life and participation only occurred after significant changes in aspects of neurodisability had become established. These results have obvious implications for specifying the sequence of neurorehabilitation interventions in order to best optimize proximal and distal treatment outcomes, and relates, as has been proposed previously (e.g., Walsh et al., 2014), to the need for interventional integration across the physical, psychological and social aspects of the person’s experience.

## Conflict of Interest Statement

The authors declare that the research was conducted in the absence of any commercial or financial relationships that could be construed as a potential conflict of interest.
